# Prevalence and risk factors of undernutrition among schoolchildren in the Plateau Central and Centre-Ouest regions of Burkina Faso

**DOI:** 10.1186/s40249-016-0230-x

**Published:** 2017-01-19

**Authors:** Séverine Erismann, Astrid M. Knoblauch, Serge Diagbouga, Peter Odermatt, Jana Gerold, Akina Shrestha, Grissoum Tarnagda, Boubacar Savadogo, Christian Schindler, Jürg Utzinger, Guéladio Cissé

**Affiliations:** 1 Swiss Tropical and Public Health Institute, P.O. Box, CH-4002, Basel, Switzerland; 2 University of Basel, P.O. Box, CH-4003, Basel, Switzerland; 3 Institut de Recherches en Sciences de la Santé, P.O. Box 7192, Ouagadougou 03, Burkina Faso; 4 Kathmandu University, P.O. Box 6250, 45200 Dhulikhel, Nepal

**Keywords:** Anaemia, Burkina Faso, Intestinal parasitic infections, School garden, Undernutrition, Water, sanitation, and hygiene (WASH)

## Abstract

**Background:**

Multiple factors determine children’s nutritional status, including energy and nutrient intake, recurrent infectious diseases, access (or lack thereof) to clean water and improved sanitation, and hygiene practices, among others. The “Vegetables go to School: improving nutrition through agricultural diversification” (VgtS) project implements an integrated school garden programme in five countries, including Burkina Faso. The aim of this study was to determine the prevalence of undernutrition and its risk factors among schoolchildren in Burkina Faso before the start of the project.

**Methods:**

In February 2015, a cross-sectional survey was carried out among 455 randomly selected children, aged 8–14 years, in eight schools in the Plateau Central and Centre-Ouest regions of Burkina Faso. Nutritional status was determined by anthropometric assessment. Helminth and intestinal protozoa infections were assessed using the Kato-Katz and a formalin-ether concentration method. A urine filtration technique was used to identify *Schistosoma haematobium* eggs. Prevalence of anaemia was determined by measuring haemoglobin levels in finger-prick blood samples. Questionnaires were administered to children to determine their knowledge of nutrition and health and their related attitudes and practices (KAP). Questionnaires were also administered to the children’s caregivers to identify basic household socio-demographic and economic characteristics, and water, sanitation and hygiene (WASH) conditions. To determine the factors associated with schoolchildren’s nutritional status, mixed logistic regression models were used. Differences and associations were considered statistically significant if *P*-values were below 0.05.

**Results:**

Complete datasets were available for 385 children. The prevalence of undernutrition, stunting and thinness were 35.1%, 29.4% and 11.2%, respectively. The multivariable analysis revealed that undernutrition was associated with older age (i.e. 12–14 years compared to <12 years; adjusted odds ratio (a*OR*) = 3.45, 95% confidence interval (*CI*) 2.12–5.62, *P* < 0.001), multiple pathogenic parasitic infections (a*OR* = 1.87, 95% *CI* 1.02–3.43, *P* = 0.044) and with moderate and severe anaemia in children (a*OR* = 2.52, 95% *CI* 1.25–5.08, *P* = 0.010).

**Conclusions:**

We found high prevalence of undernutrition among the children surveyed in the two study regions of Burkina Faso. We further observed that undernutrition, anaemia and parasitic infections were strongly associated. In view of these findings, concerted efforts are needed to address undernutrition and associated risk factors among school-aged children. As part of the VgtS project, WASH, health education and nutritional interventions will be implemented with the goal to improve children’s health.

**Trial registration:**

ISRCTN17968589 (date assigned: 17 July 2015).

**Electronic supplementary material:**

The online version of this article (doi:10.1186/s40249-016-0230-x) contains supplementary material, which is available to authorized users.

## Multilingual abstracts

Please see Additional file 1 for translations of the abstract into the five official working languages of the United Nations.

## Background

In Burkina Faso, undernutrition, anaemia and diarrhoeal diseases are the leading causes of morbidity in children under the age of five. The most recent Demographic and Health Survey (DHS) of 2010 showed that 88% of children under five were anaemic, 35% were undernourished and 15% suffered from diarrhoea in the two weeks preceding the DHS [1]. While DHS and national nutrition surveillance systems in Burkina Faso have routinely measured the height and weight of children under the age of five since the early 1990s, there is a lack of national anthropometric data for school-aged children (5–14 years) [2–4].

The determinants of children’s nutritional status are multifactorial [5–7]. The direct causes of undernutrition in children are insufficient energy and nutrient intake, and recurrent infectious diseases (e.g. intestinal parasitic infection, malaria and diarrhoea) [7]. Factors that affect children’s nutritional status indirectly include a lack of access to clean water and improved sanitation, inadequate hygiene, a paucity of health education and, importantly, inappropriate agricultural practices and insufficiently healthy and diverse diets [5–9]. Low socio-economic and sanitary conditions prevail in Burkina Faso and together contribute to the burden of infectious diseases in children [1, 10, 11], further compromising their nutritional status [5–9, 12].

To address these challenges, research institutions and international development organisations are paying increased attention to enhancing synergies among agriculture, nutrition and health. The Sustainable Development Goals (SDGs) have recognised agriculture as a source of nutrition and well-being, as addressed in SDG 2: “End hunger, achieve food security and improved nutrition and promote sustainable agriculture” [13]. Yet, there is a dearth of evidence to support the effect of agricultural and health interventions on improving children’s nutritional status [14, 15]. To fill this research gap, a multi-country and multi-stakeholder project entitled “Vegetables go to School: improving nutrition through agricultural diversification” (VgtS), was developed to address schoolchildren’s nutrition in an interdisciplinary way, through introducing school vegetable gardens and other school-based health, nutritional and environmental interventions. The VgtS project is active in five countries in Africa and Asia (Bhutan, Burkina Faso, Indonesia, Nepal and the Philippines), with the overall goal of improving schoolchildren’s nutritional status [16]. Under the VgtS project, two intervention studies were implemented in Burkina Faso and Nepal. These studies assessed schoolchildren’s nutritional and health status at baseline and at 12 months follow-up, using a set of selected qualitative and quantitative indicators. The findings from these studies guided the development of complementary nutrition and water, sanitation and hygiene (WASH) interventions to operate alongside the school garden programme. Details of the study design and procedures have been described elsewhere [16].

The Burkina Faso setting provided an opportunity to understand the complex interactions among agriculture, undernutrition, intestinal parasitic infections and WASH conditions. Agriculture is a major source of livelihoods in the country and inadequate WASH conditions are well known risk factors for both undernutrition and intestinal parasitic infections [11, 17–20]. In this article, we report findings from a cross-sectional baseline survey carried out in Burkina Faso as part of the intervention component of the VgtS project.

## Methods

### Study area

**Fig. 1:**
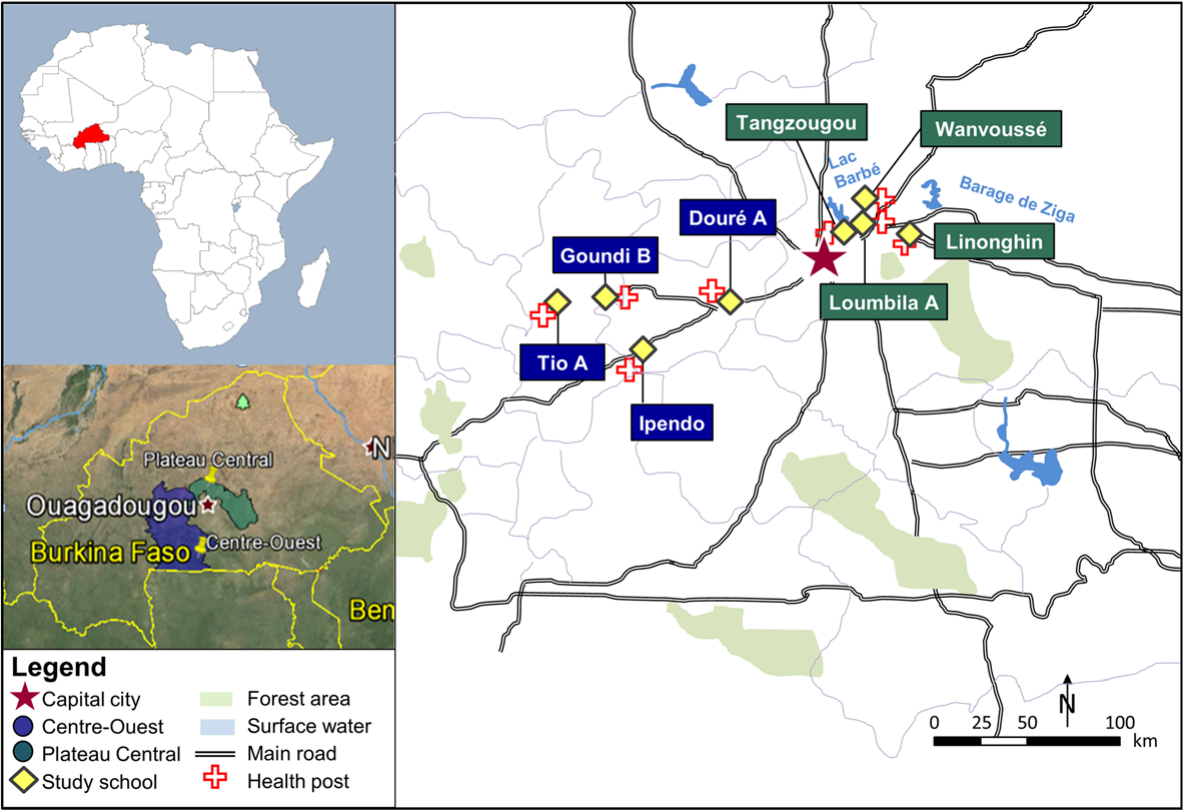
Study sites of the cross-sectional survey in Burkina Faso, February 2015

We conducted a cross-sectional baseline study in February 2015. The schools participating in the VgtS project in Burkina Faso are located in the Plateau Central and the Centre-Ouest regions. The Plateau Central region is situated in the north-east, approximately 30–120 km from the capital, Ouagadougou. The Centre-Ouest region is located in the south-west, some 40–180 km from Ouagadougou (Fig. 1). The two regions are located in the semi-arid North-Sudanian zone, characterised by fields, bushes and scattered trees and a Sudano-Sahelien climate (a short wet and a long dry season, with annual precipitation of 600–1 000 mm).

### Sample size and sampling method

Our sample size calculation targeted the association between the prevalence of intestinal parasitic infection and the degree of risk among children, aged 8–14 years. We assumed a minimum prevalence of intestinal parasitic infections of 40%, with a coefficient of variation of 10% across schools and a proportion of high - risk children of 25%. We aimed for a power of 85% to detect a difference in infection rates (with *P* < 0.05) between high- and low-risk children at eight schools, for a true odds ratio (*OR*) of at least 2. A Monte Carlo simulation (5 000 iterations) provided a minimal sample size of 400 children (i.e. 50 children per school). Eight of the 30 VgtS project schools in Burkina Faso were randomly selected to participate in the study [16]. In each of the sampled schools, 55–60 children (boys and girls in ratio 1:1) were randomly selected; we assumed that the final sample size would be reduced by 15% due to non-response and missing data [16]. The inclusion criteria for this study were: (i) schoolchildren between the ages of 8 and 14 years; (ii) parents/guardians of the children providing written informed consent; and (iii) children additionally providing oral assent.

### Anthropometric survey

Trained field staff collected anthropometric measurements from the children, using a height measuring board and a digital scale (Seca 877; Seca, Germany) with a precision of 0.1 cm and 0.1 kg, respectively and adhering to standard procedures [21]. Anthropometric indices were calculated in accordance with the World Health Organization (WHO) reference, using AnthroPlus (WHO; Geneva, Switzerland) [22, 23]. For children without an exact date of birth or whose age was unknown, school registration lists were consulted. If the exact month or date of birth was unavailable, anthropometric indices were calculated assuming 30 June (mid-year) as the child’s date of birth. Three anthropometric indices — height-for-age (HAZ, stunting), body mass index-for-age (BMIZ, thinness) and weight-for-age (WAZ, underweight) — were expressed as differences from the median in z-scores. Children were classified as stunted, thin, or underweight if z-scores of HAZ, BMIZ and WAZ were less than - 2 standard deviations (SD) below the WHO reference median of the standard population. WAZ was only used for children aged 8–10 years, as reference data were not available for children over 10 years [22, 23]. Children were classified as overweight if BMIZ was above 1 SD. We considered children to be malnourished when classified as stunted, thin, underweight or overweight; undernourished children were those classified as stunted, thin or underweight. The categories of stunting, thinness and underweight are not mutually exclusive, as these conditions often overlap; an undernourished child can, for example, be classified as stunted and thin, concurrently.

### Haemoglobin survey

Haemoglobin (Hb) concentration was determined in finger-prick capillary blood samples, using a HemoCue portable device (HemoCue Hb 201 System; Ängelholm, Sweden) [24]. Children were classified as mildly anaemic if Hb concentration was less than 11.5 g/dl for children aged 8–11 years and less than 12 g/dl for children aged 12–14 years. Children were classified as moderately and severely anaemic if Hb concentration was less than 11 g/dl and 8 g/dl, respectively [25].

### Parasitological survey

Children were asked to provide a fresh morning stool and a mid-morning post-exercise urine sample, collected on two consecutive days. Stool and urine samples were processed the same day by experienced laboratory technicians. From each stool, a single Kato-Katz thick smear was prepared for diagnosis of soil-transmitted helminths (*Ascaris lumbricoides*, hookworm and *Trichuris trichiura*), *Schistosoma mansoni* and other helminths. A formalin-ether concentration (FEC) technique was also performed on each sample to diagnose helminths and intestinal protozoa *(Blastocystis hominis*, *Chilomastix mesnili*, *Endolimax nana*, *Entamoeba coli*, *Entamoeba histolytica*/*E. dispar*, *Entamoeba hartmanni*, *Giardia intestinalis*, and *Iodamoeba bütschlii*) [26, 27]. Urine samples were examined for microhaematuria using reagent strips (Hemastix, Siemens Healthcare Diagnostics GmbH; Eschborn, Germany). A urine filtration technique was applied to detect the presence and number of *S. haematobium* eggs [28]. Helminth infection intensity was calculated based on criteria established by the WHO [29].

### Questionnaire survey

Questionnaires were administered to children to determine their knowledge of nutrition and health and associated attitudes and practices (KAP) and to the caregivers to identify basic household socio-demographic and economic characteristics and WASH conditions. The KAP and household questionnaires were established according to international guidelines, using standardised questions amended by our research team [1, 30, 31]. Both questionnaires were pre-tested in the study area in November 2014, with children and caregivers who did not subsequently participate in the survey (as part of a pilot study carried out in different schools and villages, far away from those schools selected for the present study). Final local adaptations were made prior to the start of the survey in February 2015.

### Data entry and storage

Data were double-entered in Excel 2010 (Microsoft; Redmond, USA). After removing inconsistencies, the datasets were combined and the accuracy of the merged database was verified against the original data through random cross-checking. Data were transferred to and stored electronically on a secure and password-protected server at the Swiss Tropical and Public Health Institute (Swiss TPH; Basel, Switzerland).

### Statistical analysis

Categorical variables were described by absolute and relative frequencies. Numerical variables were described by their mean and SD if they were normally distributed, and by their median and interquartile range, otherwise. To characterise household socioeconomic status, we conducted a factor analysis. A list of recorded household assets were included, which took into account the construction materials of the house wall, roof and floor [32]. Four factors reflecting four different socioeconomic domains were retained, including; (i) housing wall materials; (ii) roof materials; (iii) floor materials; and (iv) main energy sources used.

To test for associations between undernutrition (including stunting, thinness and underweight) in children as an outcome variable and associated risk factors, we first conducted a univariable mixed logistic regression analysis with random intercepts at the level of the schools. We included random effects for schools in our logistic regression models, as outcomes might vary between schools due to local factors not accounted for in our models. Non-pathogenic, intestinal protozoa infections (*Trichomonas intestinalis* and *E. coli*) were excluded as potential risk factors for undernutrition in univariable and multivariable analysis. A new variable for hygiene behaviour was created using factor analysis with two conceptually similar categorical variables of: (i) mode of handwashing (e.g. handwashing with soap and water, with water only, with ash, and no handwashing); and (ii) handwashing frequency (before eating, after eating, after playing, and after defecation). Children were classified into one of three categories, reflecting poor, moderate or better hygiene behaviours.

Second, we used a multivariable mixed logistic regression model with random school intercepts and including the categorical exposure variables sex, age, project region and household socioeconomic status as additional independent variables. All other variables were added to the core model one by one, and those with a *P* < 0.2 (using likelihood ratio test) were included in the final multivariable model. *OR*s were reported to compare relative odds, while differences and associations were considered as statistically significant if *P*-values were below 0.05, and indicating a trend if *P*-values were between 0.05 and 0.1.

Statistical analyses were performed with Stata version 13 (StataCorp; College Station, USA). Maps, including geographical coordinates of the schools, were established in ArcMap™ version 10 (Environmental System Research Institute; Redlands, USA) and with the Google Earth™ mapping software (https://www.google.com/earth).

## Results

### Study compliance and respondents’ characteristics

**Fig. 2:**
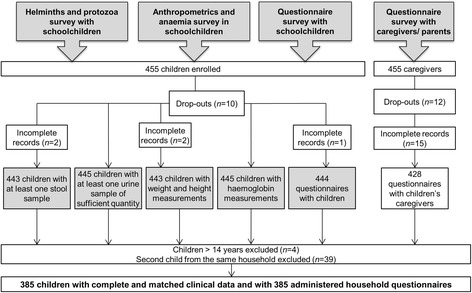
Participation in the different study groups of the cross-sectional survey in Burkina Faso, February 2015

Overall, 455 schoolchildren from eight schools were enrolled in the study. Figure 2 summarises study participation and compliance, from enrolment to the final sample included for statistical analysis.

Parasitological, anthropometric, Hb and KAP questionnaire data were linked by means of a unique identification code (ID). Erroneous ID codes or incomplete datasets with at least one of the parameters missing (e.g. anthropometrics, anaemia, urine and stool analyses, and child and household questionnaires) reduced the number of complete datasets from 455 to 424 children’s records and 385 corresponding household records for subsequent analyses. For households with more than one participating child, one child was selected at random for analysis; hence, another 39 children were excluded and our final dataset comprised 385 children from 385 unique households.

The mean age of children interviewed was 11 years (SD 0.7 years, range: 8–14 years). The mean age of the children’s caregivers interviewed was 45 years (SD 14.2 years, range: 20–95 years). Three-quarters of the children’s caregivers had not received any formal education, whereas 59 (15.3%) attended primary school and the remaining 38 (9.9%) received at least a secondary level of education. Almost 90% of children’s caregivers work in the agricultural sector. Respondents’ demographic and economic characteristics are summarised in Table 1.

**Table 1:** Characteristics of the study population in the Plateau Central and Centre-Ouest regions, Burkina Faso, February 2015

Children’s demographic characteristics		Number	Percent
Age of children^a^			
Girls		188	48.8
Boys		197	51.2
Age group 1 (8–11 year)		251	65.2
Age group 2 (12–14 years)		134	34.8
Caregivers’ demographic and educational characteristics			
Caregivers’ age^b^			
No formal schooling		288	74.8
Primary education		59	15.3
Secondary or higher education		38	9.9
Main occupation of head of household			
Agriculture		344	89.4
Merchant		8	2.1
Civil service		9	2.3
No employment		2	0.5
Others (housework or retirement)		22	5.7
Socioeconomic domains			
Roof material	Simple (natural and baked clay)	37	9.6
	Metal cover	348	90.4
Wall material	Simple (natural clay)	359	93.3
	Baked or cemented clay	26	6.7
Floor material	Simple (clay, sand, mud, straw)	255	66.2
	Baked or cemented clay	130	33.8
Energy used	Simple (charcoal, firewood)	376	97.7
	Electricity and gas	9	2.3

^a^ = mean age of 11.0 (±0.7) years

^b^ = mean age of 45.0 (±14.2) years

### Prevalence of malnutrition

Table 2 shows the extent of malnutrition, stratified by anthropometric indicators, including age, sex and region. The prevalence of malnutrition and undernutrition in this study were high, at 37.1% and 35.1%, respectively. The prevalence of stunting was 29.4%, while 11.2% of the children were classified as thin. Three out of the 55 children under the age of 10 years were underweight, while eight children were classified as overweight.

**Table 2:** Prevalence of total and specific malnutrition indicators in schoolchildren, Burkina Faso, February 2015

Variable	Malnutrition [*n* (%)]	Undernutrition [*n* (%)]	Stunting^a^ [*n* (%)]	Thinness^a^ [*n* (%)]	Underweight^a^ [*n* (%)]	Overweight^b^ [*n* (%)]	Anaemia^c^ [*n* (%)]
Sex							
Female (188)	61 (32.5)	57 (30.3)	47 (25.0)	24 (12.8)	2 (1.1)	4 (2.1)	53 (28.2)
Male (197)	82 (41.6)	78 (39.6)	66 (33.5)	19 (9.6)	1 (0.5)	4 (2.0)	57 (28.9)
Age group							
8–11 year (251)	69 (27.5)	61 (24.3)	47 (18.7)	16 (6.4)	3 (1.2)	8 (3.2)	55 (21.9)
12–14 years (134)	74 (55.2)	74 (55.2)	66 (49.3)	27 (20.2)	*NA* ^d^	0 (0)	55 (41.0)
Region							
Plateau Central (198)	69 (34.9)	64 (32.3)	50 (25.3)	19 (9.6)	2 (1.0)	5 (2.5)	53 (26.8)
Centre-Ouest (187)	74 (39.6)	71 (38.0)	63 (33.7)	24 (12.8)	1 (0.5)	3 (1.6)	57 (30.5)
Total	143 (37.1)	135 (35.1)	113 (29.4)	43 (11.2)	3 (0.8)	8 (2.1)	110 (28.6)

^a^ z-score < − 2

^b^ z-score > 1

^c^ The category of anaemia includes all children classified as anaemic (mild, moderate and severe) based on the concentrations of haemoglobin (Hb) determined in a finger prick blood sample. The cut-offs for anaemia are age-specific: Hb <11.5 g/dl for children aged 8–11 years, and Hb <12 g/dl for children aged 12–14 years

^d^ *NA* not available

### Intestinal parasitic and *Schistosoma* infections

Table 3 shows differences in the prevalence of intestinal protozoa, faecal-oral transmitted helminths and *Schistosoma* infections in children, stratified by sex, age and region. We found that 86.2% of the children were infected with at least one intestinal parasite. Intestinal protozoa infections were highly prevalent (84.7%). *Entamoeba histolytica/E. dispar* was the predominant intestinal protozoon species (66.5%), followed by *E. coli* (37.4%), *G. intestinalis* (28.1%) and *T. intestinalis* (23.4%).

**Table 3:** Prevalence of helminths and intestinal protozoa infections in schoolchildren, Burkina Faso, February 2015

Variable	Trematodes		Total schistosomiasis^a^ [*n* (%)]	Nematodes	Cestodes	Total faecal-oral transmitted helminths^c^ [*n* (%)]	Protozoa					Total protozoa [*n* (%)]
	*S. haematobium* ^*a*^ [*n* (%)]	*S. mansoni* ^a^ [*n* (%)]		Hookworm [*n* (%)]	*H. nana* ^b^ [*n* (%)]		*Entamoeba histolytica*/*E. dispar* [*n* (%)]	*Entamoeba coli* [*n* (%)]	*Giardia intestinalis* [*n* (%)]	*Trichomonas intestinalis* [*n* (%)]	*Balantidium coli* [*n* (%)]	
Sex												
Female (188)	7 (3.7)	0 (0)	7 (3.7)	0 (0)	11 (5.9)	11 (5.9)	131 (69.7)	67 (35.6)	44 (23.4)	39 (20.7)	1 (0.5)	161 (85.6)
Male (197)	8 (4.1)	1 (0.5)	9 (4.6)	3 (1.5)	14 (7.1)	16 (8.1)^c^	125 (63.5)	77 (39.1)	64 (32.5)	51 (25.9)	0 (0)	165 (83.8)
Age group												
8–11 year (251)	8 (3.2)	0 (0)	8 (3.2)	2 (0.8)	13 (5.2)	15 (6.0)	163 (64.9)	93 (37.1)	69 (27.5)	51 (20.3)	0 (0)	209 (83.3)
12–14 years (134)	7 (5.2)	1 (0.8)	8 (6.0)	1 (0.8)	12 (9.0)	12 (9.0)^c^	93 (69.4)	51 (38.1)	39 (29.1)	39 (29.1)	1 (0.8)	117 (87.3)
Region												
Plateau Central (198)	8 (4.0)	0 (0)	8 (4.0)	1 (0.5)	5 (2.5)	6 (3.0)	110 (55.6)	65 (32.8)	49 (24.8)	55 (27.8)	0 (0)	157 (79.3)
Centre-Ouest (187)	7 (3.7)	1 (0.5)	8 (4.3)	2 (1.1)	20 (10.7)	21 (11.2)^c^	146 (78.1)	79 (42.3)	59 (31.6)	35 (18.7)	1 (0.5)	169 (90.4)
Total (385)	15 (3.9)	1 (0.3)	16 (4.2)	3 (0.8)	25 (6.5)	27 (7.0)	256 (66.5)	144 (37.4)	108 (28.1)	90 (23.4)	1 (0.3)	326 (84.7)

^a^ *Schistosoma haematobium*, *Schistosoma mansoni*

^b^ *Hymenolepis nana*

^c^ The category of total faecal-oral transmitted helminths includes children infected with hookworm and *Hymenolepis nana*. There is one child co-infected with hookworm and *Hymenolepis nana.*

Faecal-oral transmitted helminth infections were found in 7.0% of the children. *Hymenolepis nana* was the most frequently occurring species (6.5%). Only three children were infected with hookworm (0.8%). One child had a dual-species infection with hookworm and *H. nana*. Fifteen children were infected with *S. haematobium* (3.9%), while one child was infected with *S. mansoni* (0.3%).

Co-infections were common, affecting 32.5% of the children, whilst 15.6% and 4.7% suffered from triple and quadruplicate infections, respectively. Infections with *H. nana*, *S. haematobium,* hookworm and *S. mansoni* were of light intensity. The prevalence of intestinal protozoa and faecal-oral transmitted helminth infections differed significantly between schoolchildren in the Plateau Central region and those in Centre-Ouest (*P* < 0.05).

### Prevalence of anaemia

The mean Hb concentration was 12.3 g/dl (SD 0.7 g/dl). The prevalence of anaemia in our study sample was 28.6% (Table 2). Few children were found to be severely anaemic (0.8%), while 11.2% were found to be moderately anaemic and 16.6% mildly anaemic.

### Results from the questionnaire surveys

Key results from children’s nutrition and health KAP survey and from the household questionnaire are summarised in Table 4. While 79.7% of the children reported using latrines at school for defecation, 22.1% reported washing their hands after defecation. Most children (87.8%) reported washing their hands before eating and 7.3% after playing. Four out of five (79.5%) children reported using soap and water to wash their hands. Combining the mode and frequency of handwashing, children were divided into one of three hygiene categories: 14.6% in the lower, 59.0% in the middle and 26.4% in the better hygiene category. Among the households participating in our survey, 55.3% did not own a latrine, while 23.1% had access to an improved latrine. The majority of children (82.1%) and 22.1% of their caregivers stated that they had never heard of malnutrition. Of the interviewed caregivers, 96.9% indicated that their participating child was breastfed.

**Table 4:** Key findings from children’s nutrition and health KAP survey and household questionnaire in Burkina Faso, February 2015

Children (*n* = 385)	Number	Percent
Selected KAP^a^ indicators:		
Handwashing^b^		
Water only	344	89.4
Water and soap	306	79.5
With ash	12	3.1
With mud	1	0.3
Before eating	338	87.8
After eating	55	14.3
After playing	28	7.3
After defecation	85	22.1
Do not wash hands	16	4.2
Hygiene behaviour^c^		
Lower category (1)	56	14.6
Middle score (2)	227	59.0
Best category (3)	102	26.4
Sanitary behaviour at school		
Using latrines at school	307	79.7
Open defecation (fields, bush)	71	18.5
Others (at home, teachers home)	7	1.8
Meals (day prior to the survey)		
Breakfast	330	85.7
Lunch	351	91.2
Dinner	358	93.0
Nutritional knowledge		
Heard about malnutrition	69	17.9
Households (*n* = 385)	Number	Percent
Household WASH^d^ characteristics		
Availability of soap (observational)	118	30.7
Type of latrines used		
Flush toilet (i)	0	0
VIP latrine^e^ (ii)	14	3.6
Traditional pit latrine (iii)	83	21.6
EcoSan^f^ (iv)	60	15.6
Samplat latrine (v)	15	3.9
No facilities/open defecation (vi)	213	55.3
Total improved^g^ (i, ii, iv, v)	89	23.1
Total unimproved^h^ (iii, vi)	296	76.9
Nutritional knowledge and practices		
Heard about malnutrition	300	77.9
Participating child was breastfed	373	96.9

^a^ Knowledge, attitudes and practices

^b^ Multiple responses occurred for the variables characterising the mode (how) and frequency (when) of handwashing.

^c^ A new variable for hygiene behaviour was created using factor analysis with two conceptually similar categorical variables of: (i) mode of handwashing (handwashing with water and soap, with water only, with ash, no handwashing); and (ii) its frequency (before eating, after eating, after playing, and after defecation). Children were classified into three categories with lower, middle and better hygiene behaviours.

^d^ Water, sanitation and hygiene

^e^ Ventilated improved pit (VIP) latrine is an improved type of pit latrine, which helps remove odours and prevent flies from breeding and escaping. Excreta are collected in a dry pit which has a vent pipe covered with a fly-proof screen at the top

^f^ Ecological sanitation (EcoSan) toilets are linked to a closed system that does not need water. The toilet is based on the principle of safely recycling excreta resources to create a valuable resource for agriculture

^g^ The total improved sanitation category includes sanitation facilities that hygienically separate human excreta from human contact. In our study, these were: (i) flush toilet, (ii) VIP latrine, (iv) EcoSan toilets, and (v) latrine with slab

^h^ The total unimproved sanitation category in our study included: (iii) traditional pit latrines, and (vi) no facilities/open defecation)

### Results from the logistic regression analysis

Table 5 provides an overview of the associations between undernutrition and all measured helminth and pathogenic intestinal protozoa infections, nutrition and health KAP, caregivers’ socioeconomic characteristics and WASH conditions observed in univariable and multivariable regression analyses. The prevalence of undernutrition significantly differed between age groups, with the older age group (12–14 years) showing significantly higher odds of undernutrition (a*OR* = 3.45, 95%* CI* 2.12–5.62, *P* < 0.001). Girls showed lower odds of being undernourished, but this association lacked statistical significance in the multivariable analysis. No significant association was observed between undernutrition and study region (*P* > 0.05).

**Table 5:** Results from univariable and multivariable logistic regression analysis with undernutrition as outcome

Undernutrition *N* = 385 / *N*(cases) = 135			Univariable logistic regression^a^			Multivariable logistic regression^b^		
			*OR*	95% *CI*	*P*	a*OR*	95%* CI*	*P*
Sex		Male	1.00					
		Female	0.70	0.45–1.09	**0.112**	0.72	0.46–1.14	0.163
Age group		8–11 year	1.00					
		12–14 years	3.57	2.20–5.78	**<0.001**	3.45	2.12–5.62	**<0.001**
Region		Centre-Ouest	1.00					
		Plateau Central	0.89	0.35–2.27	0.804			
Multiple pathogenic parasites		”yes” vs. “no”	1.94	1.09–3.47	**0.025**	1.87	1.02–3.43	**0.044**
Intestinal pathogenic protozoa		“yes” vs. “no”	1.78	1.03–3.06	**0.039**	1.71	0.97–3.03	0.064
*Hymenolepis nana*		“yes” vs. “no”	1.42	0.60–3.36	0.425			
*Schisotosoma haematobium*		“yes” vs. “no”	0.76	0.22–2.56	0.659			
*Giardia intestinalis*		“yes” vs. “no”	1.44	0.90–2.32	**0.131**	1.46	0.89–2.40	0.133
*Entamoeba histolytica/E. dispar*		“yes” vs. “no”	1.39	0.85–2.25	**0.187**	1.41	0.85–2.34	0.184
Anaemia		No	1.00					
		Mild	1.59	0.89–2.85	**0.121**	1.24	0.67–2.31	0.486
		Moderate^c^	2.89	1.48–5.64	**0.002**	2.52	1.25–5.08	**0.010**
		Middle score (2)	1.00					
Hygiene^d^		Lower category (1)	1.15	0.59–2.25	0.676			
		Best category (3)	1.36	0.82–2.25	0.233			
Sanitary behaviour at school		Open defecation^e^	1.00					
		Using latrines at school	0.97	0.48–1.95	0.922			
		Others (at teachers’)	Na					
Household sanitary conditions		Improved latrines	1.00					
		No latrines/open defecation	0.96	0.54–0.54	0.886			
		Traditional latrine	1.18	0.60–2.29	0.634			
Availability of soap		“yes” vs. “no”	1.14	0.70–1.84	0.599			
Child’s eating habits (day prior to the survey)	Breakfast	“no vs. yes”^f^	0.72	0.38–1.38	0.326			
	Lunch	“no vs. yes”^f^	1.88	0.89–4.00	**0.100**	1.52	0.69–3.32	0.298
	Dinner	“no vs. yes”^f^	1.30	0.57–2.99	0.534			
Child “heard about malnutrition”		“no vs. yes”^f^	1.11	0.64–1.95	0.709			
Caregiver “heard about malnutrition”		“no vs. yes”^f^	1.14	0.67–1.94	0.618			
“Breastfed child”		“no vs. yes”^f^	2.20	0.41–11.71	0.354			
Caregiver’s education		Never went to school	1.00					
		Primary education	1.30	0.71–2.37	0.390			
		Secondary education	0.87	0.40–1.89	0.716			
Caregiver’s occupation		Agriculture	1.00					
		Civil service	0.35	0.04–3.01	0.341			
		Merchant	0.35	0.33–5.23	0.702			
		Others^g^	0.71	0.28–1.85	0.487			

^a^ *P*-value and odds ratio (*OR*) based on likelihood ratio test. In univariable logistic regression, the overall *P*-value of the models is indicated in bold letters

^b^ *P*-value and adjusted (a) *OR* based on likelihood ratio test of the multivariable regression model. The mixed multivariable logistic regression model with random school intercepts included the categorical exposure variables sex, age group, socioeconomic domains and project region. All risk factors that had a *P*-value lower than 0.2 in the univariable analyses were included into the multivariable regression analysis (as indicated in the table)

^c^ The category of moderate anaemia includes the severely anaemic children (*n* = 3)

^d^ This variable was created with two conceptually similar categorical variables of: (i) mode of handwashing (handwashing with soap and water, with water only, with ash, no handwashing); and (ii) handwashing frequency (before eating, after eating, after playing, and after defecation) where multiple responses were possible. Children were classified into one of three categories, with lower, middle and better hygiene behaviours

^e^ Open defecation includes the category of defaecating in the bush and behind the latrines

^f^ The reference category for the *OR* is “yes” as compared to “no”

^g^ ‘Others’ includes homemakers, retirees and unemployed people

Children infected with multiple pathogenic parasites and those with moderate - to - severe anaemia, were at significantly higher odds of being undernourished (a*OR* = 1.87, 95% *CI* 1.02–3.43, *P* = 0.044; and a*OR* = 2.52, 95% *CI* 1.25–5.08, *P* = 0.010, respectively).

Overall, children with better hygiene behaviours (third category) did not show lower odds for undernutrition than those in the middle or lower hygiene categories (*P* > 0.5). Relying on traditional pit latrines or having no toilet facility at home was not associated with increased odds for undernutrition in children. Moreover, children who reported not having eaten lunch the day prior to the survey and children who were not breastfed showed higher odds of undernutrition, but these associations were not statistically significant (*P* > 0.05). Neither the level of education of the children’s caregivers nor their occupation showed any statistically significant association with undernutrition.

## Discussion

This paper presents findings from a cross-sectional survey on the prevalence of undernutrition and associated risk factors among schoolchildren, aged 8–14 years, from eight schools in the Plateau Central and Centre-Ouest regions of Burkina Faso. We found that undernutrition was highly prevalent among the surveyed children. Approximately a third of the children were undernourished (35.1%).

According to a study conducted in Ouagadougou in 2008/09 for the WHO’s “Nutrition Friendly School Initiative” (NFSI), the prevalence of stunting in schoolchildren (mean age of 11.5 years) was 8.8%, which is considerably lower than the prevalence of stunting among schoolchildren found in this study (29.4%) [33]. The proportion of thinness in children in our study was 11.2%, which is, however, comparable with the 13.7% found in the NFSI study [33]. Overweight children accounted for 2.1% of all children, with a higher incidence among children aged 8–11 years than among the older age group (3.2% vs. 0%), which is similar to the 2.3% reported in the NFSI study [33].

While few children were classified as thin, a considerably higher proportion of children in our study were stunted. Thinness is often associated with short-term risk factors, like seasonal climatic variations (which cause food scarcity/shortages) and increased occurrence of illnesses [34]. Our study was conducted in the post-harvest (mid-dry) season (February), before the commencement of the dry season (March-June) [35], suggesting that the cause of undernutrition was mainly of a chronic nature, associated with long-term risk factors.

The findings from multivariable mixed logistic regression analyses demonstrated a considerably higher risk of undernutrition among children older than 12 years of age. These results are in accordance with other studies, showing a higher prevalence of stunting in older children in low-income countries in Asia and Africa [36–38]. Moreover, children with moderate and severe anaemia (combined category) and with multiple helminths and intestinal pathogenic protozoa infections (“multiple pathogenic parasites”) showed significantly higher odds for undernutrition. Undernutrition and intestinal parasitic infections are intrinsically linked. While undernutrition and inadequate dietary intake lead to weight loss and weakened immunity and render a child more susceptible to infections, parasitic infections contribute to growth stunting by causing a vicious cycle of reduced food intake (loss of appetite), diarrhoea, malabsorption and/or increased nutrient wastage [39–41]. The observed association was statistically significant in our study, reinforcing evidence of the frequent coexistence of these conditions among children [40]. Moreover, while anaemia contributed to higher odds of undernutrition among children in our study, the aetiology of anaemia is multifactorial and can result from nutritional deficiencies and parasitic infections, among other things, which have been closely connected to the nutritional status of African schoolchildren [42–45].

Our questionnaire survey revealed important inadequacies in nutrition- and health-related knowledge and practices, but no clear association between undernutrition and WASH conditions or nutritional and health KAPs.

Our study has three main limitations. First, the findings presented here cannot be generalised for all of Burkina Faso. Despite the random selection of schools with a sample size large enough for children in this age range, the results are only representative of two regions. Second, the anthropometric survey has certain limitations with respect to the inaccuracy of children’s dates of birth. Indeed, we noted that a considerable number of children had their birthdays either on 31 December or on 1 January, according to the existing school records. Upon further probing in the interview, the children often did not know their exact date of birth. Hence, for these children, we took a mid-year point as the date of birth [46]. Third, only one single Kato-Katz thick smear and FEC from two stool samples from two consecutive days were examined for each participant. Our results may therefore underestimate the true prevalence of parasitic infections, due to the low sensitivity of the Kato-Katz technique and urine concentration method [47, 48].

Despite these limitations, our findings highlight a number of important issues. First, undernutrition in schoolchildren in this part of Burkina Faso is highly prevalent. We therefore suggest giving greater attention to the overall nutritional status of school-aged children. So far, comprehensive population-based data, such as the DHS, focus on adolescents over the age of 15 years for sexual and reproductive health issues or on children under 5 years of age, as they are more vulnerable and prone to disease, illness and death [1, 49–51]. Children under five are often the primary focus of strategies and actions to address malnutrition [7, 52, 53]. Despite the increased odds of survival for children after the age of five (they generally have a lower prevalence of infections when compared to children under the age of five), school-aged children have increased nutritional needs to support the adolescent growth spurt, requiring diets rich in energy and micronutrients and sufficient in both quantity and quality [54]. It is therefore crucial to address the nutritional needs of children in this age group to match their growth requirements [55].

Second, the results of our study highlight the need for a more profound understanding of how helminths and other intestinal parasites mediate pathways to undernutrition. In particular, it is important to investigate other primary factors related to the burden of undernutrition among school-aged children, such as malaria and other parasitic infections, and the bioavailability and absorption of micronutrients so as to prevent long-term effects of undernutrition [56–58].

To address the factors underlying and contributing to schoolchildren’s nutritional status, we support the growing recommendation from several agencies to enhance multidisciplinary strategies and programmes, including nutrition and WASH interventions for school-aged children, in order to ensure optimal health, growth and development continuing after the age of five [59–61]. Such measures should be reflected in the current development of targets and indicators for reaching SDG 2.

## Conclusions

This study provides new insight into the burden of undernutrition and its risk factors among schoolchildren in Burkina Faso, a country that lacks data on the health of children, aged 8–14 years. Our study shows that undernutrition is highly prevalent in the eight schools of the Plateau Central and Centre-Ouest regions (32.3 and 38.0%, respectively) of Burkina Faso. We also observed that undernutrition, anaemia and parasitic infections were strongly associated. In view of these findings, concerted efforts are needed to address undernutrition and the associated risk factors among school-aged children. As part of the VgtS project, WASH, health education and nutritional interventions will be implemented with the goal of improving schoolchildren's health.

## Additional file

Additional file 1: Multilingual abstracts in the five official working languages of the United Nations. (PDF 661 kb)

## Abbreviations

a*OR*: Adjusted odds ratio
BMIZ: Body mass index-for-age
*CI*: Confidence interval
DHS: Demographic and Health Survey
EKNZ: Ethikkommission Nordwest- und Zentralschweiz
FEC: Formalin-ether concentration
HAZ: Height-for-age
Hb: Haemoglobin
ID: Identification code
IRSS: Institute for Health Sciences Research
KAP: Knowledge, attitudes and practices
NFSI: Nutrition Friendly School Initiative
SD: Standard deviation
SDGs: Sustainable Development Goals
Swiss TPH: Swiss Tropical and Public Health Institute
VgtS: Vegetables go to School: improving nutrition through agricultural diversification
WASH: Water, sanitation and hygiene
WAZ: Weight-for-age
WHO: World Health Organization
