# Supplementary material for: Prevalence and risk factors of undernutrition among schoolchildren in the Plateau Central and Centre-Ouest regions of Burkina Faso
Source: Infect Dis Poverty. 2017 Jan 19;6:17. doi: 10.1186/s40249-016-0230-x (PMC5244543; doi:10.1186/s40249-016-0230-x)
Supplement: Additional file 1: — Multilingual abstracts in the five official working languages of the United Nations. (PDF 661 kb) [file 40249_2016_230_MOESM1_ESM.pdf]

Translation of the abstract into the five official working languages of the United Nations

انتشار نقص التغذية وعوامل خطر الإصابة به بين أطفال المدارس في مناطق الهضبة الوسطى و المركز الغربي في بوركينا فاسو

سيفيران ايرسمان, استريد م. كنوبلاوش, سيرج داكوكبا, بيتر اوديرمات, جانا جيرولد, اكينا شريسثا, تارناجدا جريسوم, بو بكر سافادوكو, كريستيان شيندلر, يورغ اوتسينغر, غويلاديو سيسيه

الخلاصة

المعلومات الأساسية: هنالك عوامل متعددة تحدد الوضع الغذائي للأطفال، بما في ذلك الطاقة والمتناول الغذائي، والأمراض المعدية المتكررة، و امكانية الحصول (أو عدم الحصول) على المياه النظيفة وخدمات الصرف الصحي المتطورة ، و اساليب النظافة الشخصية، و غيرها من الامور الاخرى. يهتم مشروع " الخظراوات تذهب الى المدرسة: لتحسين التغذية من خلال التنويع الغذائي" (VgtS) بتطبيق برنامج حديقة مدرسية متكاملة في خمس بلدان, بما فيها بوركينا فاسو. وكان الهدف من هذه الدراسة هو تحديد مستوى انتشار نقص التغذية وعوامل خطر الإصابة به بين أطفال المدارس في بوركينا فاسو قبل بدء المشروع.

الأساليب: في فبراير عام 2015، أجري استقصاء مقطعي شمل 455 طفلاً تم اختيارهم بشكل عشوائي، تتراوح أعمارهم بين 8-14 سنة، من ثماني مدارس في مناطق الهضبة الوسطى المركزالغربي في بوركينا فاسو. تم تحديد الحالة الغذائية عن طريق تقييم قياس حجم الجسم البشري. و عن طريق استخدام طريق كاتو-كاتز و طريقة تركيز الفورمالين-ايثر جرى تقييم الديدان الطفيلية و التهابات البروتوزوا المعوية. و تم استخدام تقنية ترشيح البول من أجل تحديد بيض البلهارسيا البولية. وحددت معدلات انتشار فقر الدم من خلال قياس مستويات الهيموجلوبين في عينات الدم الماخوذة عن طريق وخز الاصابع.

تم توزيع استبيانات على الأطفال لتحديد معرفتهم بالتغذية والصحة ومواقفهم وممارساتهم ذات الصلة (المواقف والممارسات). كما قدمت استبيانات لمقدمي الرعاية للأطفال لتحديد الخصائص الاجتماعية والديموغرافية والاقتصادية الأساسية للأسر، وحالة المياه والصرف الصحي والنظافة الصحية (المياه والصرف الصحي).واستخدمت نماذج ارتداد لوجستي مختلفة لتحديد العوامل المرتبطة بالحالة الغذائية لاطفال المدارس. واعتبرت الاختلافات والعوامل المرتبطة على انها ذات اهمية إحصائية إذا كانت قيمة الاحتمالية أقل من 0.05.

النتائج: كانت البيانات الكاملة متوفرة لـ 385 طفل. وكان معدل انتشار نقص التغذية بنسبة 35.1٪، ومعدل التقزم 29.4٪ ومعدل النحافة بنسبة 11.2٪. وقد أظهرت التحليل ذات المتغيرات المتعددة بأن نقص التغذية كان مرتبطا مع الكبر في العمر (أي ان 12-14 سنة مقارنة مع <12 سنة، نسبة الارجحية = 3.5، المهلة الزمنية الممنوحة للثقة 95٪ 1،2-6،5، قيمة الاحتمالية <0.001)، التهابات طفيلية مرضية متعددة = 1.9، 95٪ المهلة الزمنية الممنوحة للثقة= 1،0-4،3 و قيمة الاحتمالية= 0.044) و لحالات فقر الدم الحاد والمتوسط لدى الاطفال (نسبة الارجحية = 2.5، 95٪ 1،5-3،1٪ المهلة الزمنية ، قيمة الاحتمالية= 0.010)

الاستنتاجات: لقد وجدنا بأن معدل انتشار سوء التغذية مرتفع بين الأطفال الذين شملهم المسح في كلتا مناطق الدراسة.كما لاحظنا كذلك بأن نقص التغذية وفقر الدم الأمراض الطفيلية مرتبطه بقوة مع بعضها. في ضوء هذه النتائج، لا بد من تضافر الجهود لمعالجة نقص التغذية والعوامل المرتبطة بخطر الاصابة للأطفال في سن المدرسة. وسيتم تنفيذ التعليم الصحي و المداخلات الغذائية بهدف تحسين صحة الأطفال كجزء من مشروع الخظراوات تذهب الى المدرسة VgtS""، و مشروع المياه والصرف الصحي والنظافة "WASH".

Translated from English version into Arabic by Muthanna, through

TRANSLATORS
WITHOUT BORDERS

布基纳法索高原中部大区和中西大区的学龄儿童营养不良状况及危险因素

Séverine Erismann, Astrid M. Knoblauch, Serge Diagbouga, Peter Odermatt, Jana Gerold, Akina

Shrestha, Tarnagda Grissoum, Boubacar Savadogo, Christian Schindler, Jürg Utzinger, Guéladio Cissé

摘要：

引言：多种因素影响儿童的营养状况，如能量和营养摄取、反复感染、无法获取清洁水和卫生厕所、卫生行为等。“蔬菜进学校：通过农业多样化改善营养”项目在布基纳法索等五个国家采取综合的学校花园计划。本研究旨在调查布基纳法索学龄儿童在项目实施前的营养不良状况及危险因素。

方法：2015 年 2 月，在布基纳法索高原中部大区和中西大区的 8 所学校开展了一项横断面调查，随机抽取了 455 名 8-14 岁儿童。通过人体测量评价营养状态。通过加藤厚涂片法和福尔马林-乙醚浓缩法分别检测蠕虫和肠道原虫感染情况，通过尿液过滤法检测埃及血吸虫虫卵。通过指尖血测定血红蛋白水平判定贫血状况。通过问卷调查获取学生关于营养和卫生的知识以及相关的态度和行为。同时，对儿童的监护人进行问卷调查了解家庭基本的社会人口学和经济学特征以及水、厕所和个人卫生条件。采用混合 logistic 回归模型分析学龄儿童营养状况相关的因素。P 值小于 0.05 被认为差异和相关性具有统计学意义。

结果：385 名儿童具有完整的数据。营养不良、生长迟缓和消瘦分别为 35.1%、29.4%和 11.2%。多变量分析显示营养不良与高年龄（如 12-14 岁与 12 岁以下相比，调整的 OR 为 3.5，95%可信区间为 2.1-5.6， $P < 0.001$）、多重寄生虫感染（调整的 OR 为 1.9，95%可信区间为 1.0–3.4，$P = 0.044$）及中重度贫血（调整的 OR 为 2.5，95%可信区间为 1.3–5.1，$P = 0.010$）有关。

结论：本研究发现调查的两个区域儿童营养不良发生率较高，而且营养不良、贫血和寄生虫感染具有强相关。鉴于以上发现，需要加强协作以解决学龄儿童的营养不良及相关的危险因素。作为“蔬菜进学校“项目的一部分，为改善儿童的身体健康需要改善水、厕所和个人卫生、加强健康教育和营养干预。

Translated from English version into Chinese by Men-Bao Qian, through

CHINESE CENTER FOR DISEASE CONTROL AND PREVENTION
NATIONAL INSTITUTE OF PARASITIC DISEASES

Prévalence et facteurs de risque de la dénutrition chez les enfants d'âge scolaire dans les régions du Plateau central et du Centre Ouest du Burkina Faso

Séverine Erismann, Astrid M. Knoblauch, Serge Diagbouga, Peter Odermatt, Jana Gerold, Akina Shrestha, Tarnagda Grissoum, Boubacar Savadogo, Christian Schindler, Jürg Utzinger, Guéladio Cissé

Résumé

Contexte : De nombreux facteurs déterminent l'état nutritionnel des enfants, y compris l'apport énergétique et nutritionnel, les maladies infectieuses récurrentes, l'accès (ou son absence) à l'eau propre et à un assainissement amélioré, et les pratiques d'hygiène, entre entres. Le projet 'Légumes vont à l'école : améliorer la nutrition par la diversification agricole' (VgtS) met en œuvre un programme intégré de maternelle dans cinq pays, y compris le Burkina Faso. L'objectif de cette

étude était de déterminer la prévalence de la dénutrition et de ses facteurs risque chez les enfants d'âge scolaire au Burkina Faso avant le début du projet.
Méthodes : En février 2015, une enquête transversale a été menée auprès de 455 enfants choisis au hasard, âgés de 8-14 ans, de huit écoles dans les régions du Plateau central et Centre-Ouest du Burkina Faso. L'état nutritionnel a été déterminé par une évaluation anthropométrique. Les infections à helminthes et protozoaires intestinaux ont été évaluées en utilisant le Kato-Katz et une méthode de concentration formol-éther. Une technique de filtration de l'urine a été utilisée pour identifier les œufs de Schistosome haematobium. La prévalence d'anémie a été déterminée en mesurant les niveaux d'hémoglobine dans les échantillons de sang par piqûre au doigt. Des questionnaires ont été donnés aux enfants pour déterminer leur connaissance en nutrition et santé, et leurs attitudes et pratiques connexes (KAP). Les questionnaires ont été également donnés aux soignants des enfants pour identifier les caractéristiques sociodémographiques et économiques des foyers, et les conditions de l'eau, de l'assainissement et de l'hygiène (WASH). Pour déterminer les facteurs associés à l'état nutritionnel des écoliers, des modèles mixtes logistiques de régression ont été utilisés. Les différences et les similitudes ont été considérées comme statistiquement significatives si les valeurs p étaient inférieures à 0,05.
Résultats : Des séries de données complètes étaient disponibles pour 385 enfants. La prévalence de dénutrition, de retard de croissance et de maigreur était de 35,1% ; 29,4% et 11,2% respectivement. L'analyse multivariée a révélé que la dénutrition était liée à l'âge (c.-à-d. 12–14 ans par rapport à <12 ans ; odds ratio corrigé (*ORc*) = 3,5 ; 95% intervalle de confiance (*IC*) 2,1–5,6, *P* < 0,001), infections multiples pathogènes parasitaires (*ORc* = 1.9, 95%*IC* 1,0–3,4 ; *P* = 0,044) et avec anémie modérée et sévère chez les enfants (*ORc* = 2,5 ; 95%*0IC* 1,3–5,1 ; *P* = 0,010).
Conclusions : Nous avons constaté une forte prévalence de dénutrition chez les enfants interrogés dans les deux régions étudiées. Nous avons également observé que la dénutrition, l'anémie et les infections parasitaires étaient fortement reliées. Compte tenu de ces résultats, des efforts concentrés sont nécessaires pour traiter la dénutrition et les facteurs risques qui y sont associés chez les enfants d'âge scolaire. Dans le cadre du projet VgtS, WASH, des interventions d'éducation sanitaire et nutritionnelles seront mises en œuvre dans le but d'améliorer la santé des enfants.

Translated from English version into French by ishaklamia, through

TRANSLATORS
WITHOUT BORDERS

Распространенность и факторы риска недостаточности питания среди школьников в плато Центральной и Центр-Запад районах Буркина-Фасо

Séverine Erismann, Astrid M. Knoblauch, Serge Diagbouga, Peter Odermatt, Jana Gerold, Akina Shrestha, Tarnagda Grissoum, Boubacar Savadogo, Christian Schindler, Jürg Utzinger, Guéladio Cissé

Реферат: Несколько факторов определяют нутритивный статус детей, в том числе энергии и

потребления питательных веществ, рецидивирующие инфекционные заболевания, доступ (или его отсутствие) к чистой воде и улучшенным системам санитарии и гигиены, в частности. “Овощи идут в школу: улучшение питания за счет диверсификации сельского хозяйства” проекта (VgtS) реализует комплексную программу школа-сад в пяти странах, включая Буркина-Фасо. Целью данного исследования было определить распространенность недостаточности питания и ее факторов риска среди учащихся в Буркина-Фасо до начала проекта.

Методы: В феврале 2015 года, кросс-секционное исследование было проведено среди 455 случайно выбранных детей в возрасте 8-14 лет, в восьми школах в Центральном и Центр-Запад плато регионов Буркина-Фасо. Состояние питания определяется с помощью антропометрических оценок. Гельминтов и кишечных инфекций простейших оценивались с помощью Като-Кац и формалин-эфирного метода концентрации. Метод фильтрации мочи была использована для определения к schistosoma haematobium яйца. Распространенность анемии была определена путем измерения уровня гемоглобина в образцах крови по finger-prick. Анкетирование проходило на детей с целью определения их осведомленности по вопросам питания и здоровья и их родственные отношения и практики (ЗОП). Анкеты были также переданы опекунам детей для выявления основных бытовых социально-демографические и экономические характеристики, и воды, санитарии и гигиены (Wash) условия. Определить факторы, связанные с питанием школьников, были использованы смешанные модели логистической регрессии. Различия и ассоциаций считались статистически значимыми при p-значения меньше 0,05.

Результатаы: Полные наборы данных были доступны для 385 детей. Распространенность недоедания распространенность низкорослости и худобы составил 35,1%, 29.4% и 11,2%, соответственно. При многофакторном анализе выявлено, что недоедание было связано с возрастом (т. е. 12-14 лет <12 лет; скорректированное отношение шансов (СОШ) = 3.5, 95% доверительный интервал (ди) 2.1–5.6, *P* < 0,001), несколько патогенных паразитарных инфекций (аор = 1.9, 95%ди 1.0–3.4, *P* = 0,044) и с умеренной и тяжелой анемией у детей (аор = 2.5, 95%ди 1.3–5.1, *P* = 0,010).

Заключение: Мы обнаружили высокую распространенность недоедания среди детей, обследованных в двух обследованных регионах. Кроме того, мы отметили, что недостаточное питание, анемия и паразитарные инфекция были прочно связаны. С учетом этих результатов, необходимы совместные усилия для решения недоедания и связанных с ними факторов риска среди детей школьного возраста. В рамках проекта Vgts, WASH, воспитание здоровья и лечебное питание будут осуществляться с целью улучшения здоровья детей.

Translated from English version into Russian by Hao-Qi Zhang

Prevalencia y factores de riesgo de desnutrición entre estudiantes en el Altiplano Central y regiones del Medio Oeste de Burkina Faso

Séverine Erismann, Astrid M. Knoblauch, Serge Diagbouga, Peter Odermatt, Jana Gerold, Akina Shrestha, Tarnagda Grissoum, Boubacar Savadogo, Christian Schindler, Jürg Utzinger, Guéladio Cissé

Resumen

Antecedentes: Múltiples factores determinan el estado nutricional de los niños, incluyendo la ingesta de energía y nutrientes, enfermedades infecciosas recurrentes, acceso (o falta de acceso) a agua limpia y prácticas sanitarias y de higiene mejoradas, entre otros. Los "Vegetales van a la Escuela: Mejorando la nutrición mediante diversificación de la agricultura" el proyecto (VgtS) (por sus iniciales en inglés *Vegetables go to School*) implementa un programa de escuelas en cinco países, incluyendo Burkina Faso. El objetivo de este estudio fue determinar la prevalencia de desnutrición y sus factores de riesgo entre estudiantes en Burkina Faso antes de iniciar el proyecto.

Métodos: En febrero del 2015, se realizó una encuesta transversal entre 455 niños seleccionados aleatoriamente, de entre 8-14 años de edad, en ocho escuelas en el Altiplano Central y regiones del Medio Oeste de Burkina Faso. El estado de nutrición se determinó por valoración antropométrica. Helminto e infecciones intestinales protozoarias se evaluaron utilizando el método de Kato-Katz y un método de concentración de éter formalina. Se utilizó una técnica de filtración de orina para identificar huevos de *Schistosoma haematobium*. Se determinó la prevalencia de anemia al medir niveles de hemoglobina en muestras de sangre por punción de dedo. Se administraron cuestionarios a niños para determinar su conocimiento de nutrición y salud y sus actitudes y prácticas al respecto (KAP (*Knowledge Attitudes and Practices*). También se entregaron cuestionarios a los encargados de los niños para identificar características socio demográficas básicas del hogar y económicas, y condiciones de agua, sanidad e higiene (WASH) (*Water, Sanitation and Hygiene*). Para determinar los factores asociados con el estado nutricional de los estudiantes, se utilizaron modelos de regresión logística mixtos. Las diferencias y asociaciones se consideraron estadísticamente relevantes si los valores p- estaban por debajo de 0.05.

Resultados: Estuvieron disponibles conjuntos de datos completos de 385 niños. La prevalencia de desnutrición, falta de crecimiento y bajo peso fue de 35.1% 29.4% y 11.2%, respectivamente. El análisis con múltiples variables reveló que la desnutrición está asociada con más edad (es decir 12-14 años en comparación con <12 años; razones de probabilidad ajustada (aOR) (*adjusted Odds Ratio*) = 3.5, 95% intervalo de confianza (*CI*) 2.1–5.6, $P < 0.001$), infecciones parasitarias de múltiples patógenos (a*OR* = 1.9, 95%*CI* 1.0–3.4, $P = 0.044$) y con anemia moderada y severa en niños (a*OR* = 2.5, 95%*CI* 1.3–5.1, $P = 0.010$).

Conclusiones: Descubrimos una alta prevalencia y desnutrición entre niños encuestados en las dos regiones de estudio. Observamos además que la desnutrición, anemia e infecciones parasitarias están fuertemente asociadas. En vista de estos descubrimientos, se requieren refuerzos organizados para atender la desnutrición y los factores de riesgo asociados entre niños en edad escolar. Como parte del proyecto VgtS, WASH, se implementará educación de salud e intervenciones nutricionales con el objetivo de mejorar la salud infantil.

Translated from English version into Spanish by Alex Sanders, through

TRANSLATORS
WITHOUT BORDERS
